# Complex Presentation of Multicentric Castleman Disease with Coexisting HIV, HHV-8, and Other Opportunistic Infections

**DOI:** 10.1155/2024/8817064

**Published:** 2024-07-27

**Authors:** Yehya Tlaiss, Hadi Farhat, Firas Hasan, Rami Yazbek, Noura Shakaroun, Nizar Bitar

**Affiliations:** ^1^ University of Balamand, Balamand, Lebanon; ^2^ Lebanese University, Beirut, Lebanon

## Abstract

Castleman disease (CD) is a rare lymphoproliferative disorder characterized by benign lymph node enlargement. We present the case of a 43-year-old male with a complex medical history, including Crohn's disease treated with Adalimumab and later complicated with tuberculosis (TB) infection. Subsequently, in May 2021, he was diagnosed with human immunodeficiency virus (HIV) and started on antiretroviral therapy (efavirez, emricitabine, and tenofovir). Despite stropping adalimumab, anti-Tb, and antiviral therapy, he experienced persistent fever, neurological symptoms, and lymphadenopathy. Toxoplasmosis, Cytomegalovirus (CMV), and Human Herpesvirus-8 (HHV-8) were diagnosed and then treated. Furthermore, the patient displayed intermittent febrile episodes, pancytopenia, altered coagulation parameters, hypoalbuminemia, edema, and generalized abdominal pain, as well as radiological evidence of hepatosplenomegaly and pulmonary infiltrates. Left axillary lymph node biopsy (ALNB) was done and confirmed multicentric castleman disease (MCD). Moreover, the bone marrow aspirate showed plasmocytes. His treatment included chemotherapy with doxorubicin and rituximab while continuing his anti-Tb and antiretroviral therapy. This complex case highlights the diagnostic challenges of managing CD in the presence of multiple coexisting conditions, emphasizing the need for comprehensive evaluation in complex clinical presentations.

## 1. Introduction

Castleman disease (CD), alternatively referred to as angiofollicular lymphadenopathy or giant lymph node hyperplasia, is a rare lymphoproliferative disorder that can manifest with or without constitutional symptoms. This disease entity was initially documented in 1956 by Castleman et al. as a benign enlargement of hyperplastic lymph nodes [1]. Subsequently, in 1970, Flendrig identified two distinct histologic features of the disorder [2]. Building on these findings, Keller et al. in 1972 classified CD into two types: hyaline-vascular and plasma-cell [2]. Moreover, there is a mixed variant observed in some patients. Clinically, CD can present either as a localized or multicentric form. There are three main clinical types of CD: unicentric (localized) and two types of multicentric, which are Kaposi sarcoma-associated herpesvirus and idiopathic. The localized form is more common, with approximately 90% of cases being of the hyaline-vascular type. Patients with this type typically present with a solitary mass, and their disease typically follows a benign course [3]. On the other hand, most cases of the multicentric form are of the plasma-cell type. MCD is a polyclonal B-cell disorder characterized by intermittent flares of inflammatory symptoms and signs which include fever, sweats, fatigue, cachexia, edema, lymphadenopathy, and hepatosplenomegaly. Lab abnormalities include anemia, thrombocytopenia, hypoalbuminemia, hyponatremia, and elevated C-reactive protein or erythrocyte sedimentation rate. The multicentric form tends to be more aggressive and can lead to fatal outcomes due to infectious complications or malignancies, such as lymphoma, Kaposi sarcoma, or follicular dendritic cell sarcoma; malignancies have been reported in up to 32% of patients with MCD [4]. Both hyaline-vascular and plasma-cell types of CD typically trigger a systemic inflammatory response, and the clinical course can also be complicated by acquired systemic amyloidosis [5]. MCD can convert to lymphoma regardless of HIV status. Conversely, localized CD of the plasma-cell type has been known to co-occur with Hodgkin's disease, particularly in the same anatomical region. Among HIV-positive patients, MCD is associated with Human Herpesvirus-8 (HHV-8), also known as Kaposi sarcoma-associated herpesvirus. A study revealed a significantly elevated incidence of Kaposi sarcoma-associated, HHV-8-related non-Hodgkin's lymphoma in HIV-positive patients with MCD compared to the general HIV-positive population [4]. CD most commonly occurs in the thorax, with the neck being the second most frequent site, albeit with limited reported cases. This disease exhibits a male predilection and typically afflicts individuals in their third to fifth decades of life, with the youngest documented case being diagnosed at just 6 weeks of age. In the pediatric population, where the incidence is low, outcomes appear to be more favorable compared to adults [6]. This paper aims to describe a case of MCD, its diagnostic tools, and management.

## 2. Case Presentation

In 2020, a 43-year-old man presented to our clinic with a complex medical history. He had been diagnosed with Crohn's disease and was receiving appropriate treatment for it. However, his clinical presentation had evolved over the previous two years, marked by intermittent febrile episodes and persistent generalized fatigue. History goes back to April 2019, when the patient received surgical treatment for a perianal abscess. After 8 months, there was no closure due to the patient not adhering to the treatment. Colonoscopy was then opted for by his surgeon, whereby Crohn's disease was first diagnosed, and the patient was given Adalimumab. A computer tomography (CT) scan of the chest done in July 2020 for fever, sweating, weight loss, and cough revealed pulmonary infiltrates with a tree-in-bud pattern in the right lower lobe, alongside a 6 mm posterior segment pleural base nodule and multiple mediastinal lymph nodes, 19 mm being the biggest measure with no pleural effusion. This was presumed to be tuberculosis based on the history and imaging. Thus, the patient underwent 6 months of treatment. Following that, the patient presented to the emergency room with severe bloody diarrhea on May 2021, where he was found to be HIV positive on admission. The patient was started on antiretroviral therapy (efavirez, emricitabine, and tenofovir). However, he was still having episodes of high-grade fever despite wide spectrum coverage, and the patient was discharged against medical advice.

On October, 2021, the patient presented again complaining of progressive diffuse abdominal pain and painless generalized lymphadenopathy. Upon initial evaluation, laboratory tests revealed a constellation of abnormalities, including pancytopenia, altered coagulation parameters, hypoalbuminemia, and elevated acute phase reactants (Table 1).

Brain MRI showed a round lesion with a size of 1.2 × 1.1 cm which was suspected to be secondary to infection. Workup for toxoplasmosis, CMV, and HHV-8 was performed, including various cultures to detect Mycobacterium species. Serologic testing confirmed the presence of HHV-8, and polymerase chain reaction tests from the cerebrospinal fluid for toxoplasmosis and CMV both returned positive results. Consequently, the patient was prescribed a treatment regimen consisting of pyrimethamine 50 mg/day orally, sulfadiazine 1–1.5 g/day orally, and folinic acid 10 mg/day orally until immune reconstitution is achieved. Additionally, the patient received ganciclovir 300 mg IV every 12 hours for 2 weeks.

CT scans of the chest, abdomen, and pelvis further unveiled hepatosplenomegaly and an increase in mediastinal and axillary lymphadenopathy with bilateral retroperitoneal and iliac lymphadenopathy. An excisional biopsy of the left axillary lymph node was subsequently performed, revealing findings consistent with the plasma cell type of MCD and bone marrow aspirate showed plasmocytes.

All these findings were present while the patient was compliant with antiretroviral therapy with a viral load of 183 copies/ml. The patient was kept on antiretroviral therapy and started chemotherapy with doxorubicin and rituximab. Doxorubicin was administered at a dose of 40 mg/m^2^ IV, and rituximab at a dose of 375 mg/m^2^ IV. The chemotherapy regimen consisted of eight cycles, with each cycle repeated every three weeks. With the presence of pancytopenia and a CD4 count of 33, the patient responded well to treatment and recovered. After the chemotherapy sessions, follow-up of the patient showed remarkable lab improvement and CD4 count back to the normal range (Table 2).

## 3. Discussion

In this article, we have presented a case involving a 43-year-old male patient who exhibited seropositivity for both HIV and HHV-8, indicative of MCD. Typically, individuals diagnosed with CD fall within the age range of their 30s to 40s, categorizing them as middle-aged [7]. Our patient's age, being in his 40s, aligns well with the prevailing epidemiological data for this condition. It is noteworthy to mention that while the unicentric type of CD displays a slight female predilection, the multicentric type tends to exhibit a slight male predominance. Furthermore, the age distribution pattern for CD is bimodal [8, 9].

MCD in HIV-positive individuals is almost invariably linked to positive Kaposi sarcoma/HHV-8 status, although Kaposi sarcoma/HHV-8 can also manifest in HIV-negative patients. The pathogenesis of MCD is notably intertwined with HHV-8 and HIV infections [10]. One prevailing explanation involves the development of reactive hyperplastic lymphoid tissue due to chronic antigenic stimulation, particularly in the respiratory and gastrointestinal tracts. An alternative hypothesis posits that the etiology may involve a breakdown in immune regulation, marked by increased expression of the interleukin (IL)-6 gene—a cytokine with wide-ranging impacts on the immune system and hematopoiesis, closely associated with the development of multiple myeloma [7, 10]. Our patient had a history of HIV and was later found to be positive for HHV-8, and this was thought to have contributed to the severity of his symptoms.

Concerning the clinical presentation of MCD, patients with this syndrome exhibit multifocal lymph node lesions and generalized lymphadenopathy, particularly concentrated in the neck region. Additionally, more severe manifestations may include fever, night sweats, cachexia, fatigue, cytopenia, splenomegaly, weight loss, swelling, respiratory symptoms, hemophagocytic syndrome, and alterations in immunoglobulin levels, such as hypoglobulinemia or hyperglobulinemia [11–13]. It is worth noting that our case aligns with the broader literature as many of these symptoms were evident in our patient, underscoring the consistency of clinical patterns in reported cases.

Histologically, MCD can be categorized into three distinct patterns. The majority of cases, ranging from 80% to 90%, fall into the hyaline type, while approximately 10% to 20% are characterized as the plasma cell type. The remaining cases typically represent a mixed form [11, 12]. Our patient exhibited the plasma cell type of MCD which falls in the rarer category.

Given our patient's initial HIV diagnosis, opportunistic infections emerged at different disease stages. Owing to his compromised CD4 count, he contracted TB, CMV, and toxoplasmosis. Consequently, he received treatment for these opportunistic infections involving antibiotics and antiviral medications. Moreover, he maintained a regimen of antiretrovirals throughout the progression of his illness. It is noteworthy that cases of individuals with both MCD and concurrent HIV infection, accompanied by opportunistic infections, have been extensively documented in the literature, and our case aligns with these established findings [13].

Our case has followed a conventional approach of treatment that was appropriately given. With a low CD4 count of 33 and symptomatic pancytopenia, a chemotherapy regimen comprising rituximab and doxorubicin was administered. Following this treatment, a remarkable transformation was observed, with the complete resolution of the patient's symptoms and a significant improvement in his overall condition.

## 4. Conclusion

In summary, we have presented the case of a 43-year-old male with a dual medical history of HIV and Crohn's disease who subsequently encountered multiple opportunistic infections, including TB, toxoplasmosis, CMV, and HHV-8. Furthermore, he developed MCD confirmed by ALNB which was refractory to treatment with persistent anemia and thrombocytopenia. A decision was made to administer a chemotherapy regimen of doxorubicin and rituximab which led to the rapid resolution of his symptoms. This case significantly enhances the existing literature on MCD, a rare condition with diverse manifestations. MCD should be considered in the differential diagnosis, particularly in patients with HIV who develop multiple opportunistic infections despite ongoing treatment.

## Figures and Tables

**Table 1:** Initial patient labs.

Tests	Results	Reference values
Hemoglobin	7.1	14–18 g/dL
Hematocrit	24.6	42–52%
White blood cells	2.31	4–10 × 10 ^ 3/microL
Platelets	48	150–400 × 10 ^ 3/microL
Albumin	2.1	3.5–5.2 g/dL
Globulin	3.3	2–3.2 g/dL
Prothrombin time	18.9	10–13 sec
Partial thromboplastin time	36	25–35 sec
INR	1.44	1–1.3
Ferritin	4209	16.4–293.9 ng/ml
CD4	33	332–1642 cells/mm ^ 3
Viral load	183	<50 copies/ml

**Table 2:** Patient labs post chemotherapy.

Tests	Results	Reference values
Hemoglobin	14.4	13–18 g/dL
Hematocrit	41.3	40–54%
White blood cells	4.6	4–11 × 10 ^ 3/microL
Platelets	191	141–362 × 10 ^ 3/microL
CD4 count	479	332–1642 cells/mm ^ 3
CD8 count	1184	170–811 cells/mm ^ 3
Viral load	78	<50 copies/ml

## Data Availability

The underlying data supporting the results of our study are available from the corresponding author upon request for access to the datasets analyzed or generated during the study. We aim to ensure the transparency and reproducibility of our research findings, and we are committed to providing access to the data underlying our results to facilitate further exploration and validation by the scientific community.
